# Zebrafish cardiac regeneration—looking beyond cardiomyocytes to a complex microenvironment

**DOI:** 10.1007/s00418-020-01913-6

**Published:** 2020-09-14

**Authors:** Rebecca Ryan, Bethany R. Moyse, Rebecca J. Richardson

**Affiliations:** C21a, Biomedical Sciences Building, Faculty of Life Sciences, School of Physiology, Pharmacology and Neuroscience, University of Bristol, University Walk, Bristol, BS8 1TD UK

**Keywords:** Zebrafish, Heart regeneration, Microenvironment, Immune cells, Cardiomyocytes

## Abstract

The study of heart repair post-myocardial infarction has historically focused on the importance of cardiomyocyte proliferation as the major factor limiting adult mammalian heart regeneration. However, there is mounting evidence that a narrow focus on this one cell type discounts the importance of a complex cascade of cell–cell communication involving a whole host of different cell types. A major difficulty in the study of heart regeneration is the rarity of this process in adult animals, meaning a mammalian template for how this can be achieved is lacking. Here, we review the adult zebrafish as an ideal and unique model in which to study the underlying mechanisms and cell types required to attain complete heart regeneration following cardiac injury. We provide an introduction to the role of the cardiac microenvironment in the complex regenerative process and discuss some of the key advances using this in vivo vertebrate model that have recently increased our understanding of the vital roles of multiple different cell types. Due to the sheer number of exciting studies describing new and unexpected roles for inflammatory cell populations in cardiac regeneration, this review will pay particular attention to these important microenvironment participants.

## Introduction

Cardiovascular disease and myocardial infarction (MI) remain major global health burdens, and their relationship to the escalating obesity epidemic and an increasingly unhealthy lifestyle means their frequency is unlikely to decline in the coming years (Braunwald 2015). MI, often occurring as a consequence of coronary heart disease, can result in massive cardiomyocyte (CM) loss, perhaps up to 1 billion cells (Murry et al. 2006) and is often followed by fibrotic tissue and scar formation, severely limiting the functional capacity of the heart and leading to heart failure. The holy grail for cardiovascular therapeutics would be the ability to stimulate cardiac regeneration and replace the myocardium lost to cell death; however, this remains elusive and poorly understood, with regeneration in mammalian models restricted to early developmental stages (reviewed by Murry et al. 2006; Cahill et al. 2017). Current treatment options for cardiovascular disease range from pharmaceutical to transplant; however, there is no escaping that each strategy has associated drawbacks (reviewed by Hashimoto et al. 2018).

The ability to somehow stimulate the production of new CMs, for example via the use of stem cell or cell replacement therapies, has long been the ultimate goal (reviewed by Murry et al. 2006; Hashimoto et al. 2018). However, the limited success of CM-focused therapies has highlighted that these approaches are too narrow and overly focused on a single cell type. To develop an approach that could potentially stimulate endogenous regeneration or provide additional support to a CM replacement strategy, it will also be essential to harness a pro-regenerative microenvironment, widening the scope to encompass diverse non-myocytes and the extracellular milieu. To study this complexity, it seems logical to turn to a model system that offers both a natural regenerative ability and a complete in vivo system, criteria that are met (almost uniquely) by the zebrafish.

Surgical resection of the apex of the ventricle has been in use for almost twenty years to study the mechanisms involved in regenerating cardiac tissue in the adult zebrafish (Poss 2002). Additionally, genetic cell ablation models have been utilised to reduce CM number and trigger regeneration (Wang et al. 2011). More recently, the development of a cryoinjury model, whereby a liquid nitrogen-cooled probe is placed on the ventricle to induce localised cell death (Chablais et al. 2011; Schnabel et al. 2011; Gonzalez-Rosa et al. 2011), has provided a new aperture to view the resulting regeneration and is more representative of the cellular damage resulting from MI. Whichever injury model is chosen, studies into the complex injury response in this model are providing a wealth of information on the cellular and molecular mechanisms required to rebuild the heart after damage and are increasingly highlighting the need to widen the scope beyond CMs to the microenvironment surrounding these cells.

## Current therapeutic outlook

Despite the relatively simple architecture of the adult mammalian heart, repair is complex, and regeneration has proven unattainable thus far. As regeneration is currently impossible, and heart transplant is limited by practical considerations (not least the lack of donors but also the associated surgical complexities) (Yacoub 2015), the therapeutic focus is treatment rather than cure, specifically in preventing the progression of ischaemic heart disease to heart failure (Sacks et al. 2014). Cardioprotective treatment options can improve blood supply (e.g. revascularisation by thrombolysis or bypass surgery) and pharmacological interventions can decelerate cardiac remodelling (e.g. ACE inhibitors and β-blockers), whereas more advanced stages of heart failure benefit from mechanical support therapies (such as left-ventricular assist devices or cardiac resynchronisation therapy) (Reviewed by Hashimoto et al. 2018). Despite this broad range of therapeutic interventions, ischaemic heart failure and its associated adverse cardiac remodelling remain major challenges for health services worldwide.

Cell-replacement therapies (using, for example, direct application of (often exogenous) stem cells or re-differentiated CMs derived from somatic cells via induced pluripotent stem cells (iPSCs)) aim to directly address the problem of insufficient CM proliferation, which is widely considered to be the limiting factor in heart regeneration. These therapies, which involve growing vast numbers of cells in vitro (potentially more than the 1 billion CMs that may be lost following MI) and injecting them directly into the injured heart, have shown promise in numerous pre-clinical settings. However, results for this relatively new strategy are often inconsistent and fail to achieve marked improvements in cardiac function (reviewed by Müller et al. 2018; Tehzeeb et al. 2019), and studies in animal models have revealed concerning and prevalent side effects including arrhythmias and tachycardia (Shiba et al. 2016; Liu et al. 2018; and reviewed by Chen et al. 2020). These inconsistencies may be partly explained by a recent and important publication from Vagnozzi et al. (2020) which has found that direct chemical stimulation of the innate immune system produces an outcome comparable to treatment with cell therapies previously reported to be reparative in models of cardiac ischaemic injury (Vagnozzi et al. 2020). This comprehensive report has demonstrated that beneficial effects thought to result from stem cell therapies are chiefly mediated by an acute regional accumulation of specific macrophage populations and an inflammatory wound healing response triggered by the cell transplant, rather than direct proliferation of the transplanted cells. These findings are also cohesive with an emerging research trend to further delineate the role of the immune response in cardiac repair and regeneration, which will be the focus of this review.

## Cardiomyocyte proliferation

In recent years, the scant proliferative capacity of CMs has been highlighted as the major limiting factor in the process of heart regeneration following cardiac injury/MI. In the mature mammalian heart, CM proliferation is poor, and progenitors are present in low numbers (reviewed by Cahill et al. 2017; Hashimoto et al. 2018), with the post-natal heart mainly increasing in size via cardiac hypertrophy (Alkass et al. 2015). The composition of the adult myocardium is noteworthy when considering its regenerative potential. Despite the fact that approximately 90% of the myocardial mass is composed of CMs, these cells only represent ~ 30% of the total number of cells, the rest being predominantly composed of endothelial cells (~ 43%), fibroblasts (~ 20%) and leukocytes (~ 7%) (Reiss et al. 1996; Pinto et al. 2016). About 1% are CM progenitor cells (or stem cells), and this, coupled with the fact that the majority of adult CMs are quiescent, is thought to account for the fact that the adult human heart has a very minimal regenerative capacity post-injury, with CMs estimated to have an annual renewal rate ranging from 1% in early adulthood (25 years) to as little as 0.45% in later life (75 years) (Murry et al. 2006). Considering the cellular damage post-MI can be in the region of a loss of 25% of cells (Murry et al. 2006), it is clear that normal turnover and replacement operates at a substantial deficit and is insufficient to restore a functional myocardium post-infarction (Bergmann et al. 2009; Senyo et al. 2013). However, if only 30% of the total number of cardiac cells are CMs, it seems restrictive to narrow our focus for regenerative capacity to this single cell type.

Despite the absence of robust regeneration in the adult mammalian heart, cardiac regeneration is observed, to varying degrees, in neonatal mammals. Murine and porcine hearts are capable of efficient regeneration, providing the injury occurs in the first 2 days post-natally, and the magnitude of the injury is not too great (< 15%) (Porrello et al. 2011; Bryant et al. 2015; Notari et al. 2018; Ye et al. 2018; Zhu et al. 2018). Use of an adult zebrafish cryoinjury model also supports this finding that even in highly regenerative systems the potential is not inexhaustible. Injury resulting in up to 20% ventricular cell death (by area) seems to be well tolerated and completely resolved by 60-day post-injury (dpi) (Chablais et al. 2011; Bevan et al. 2020) yet increasing the injury area by 5% seems to push the regenerative capacity of the zebrafish heart beyond its limits, with scar resolution incomplete even at 130 dpi (Gonzalez-Rosa et al. 2011). Additionally, a recent report demonstrates that repeated cryoinjuries limit the regenerative ability of adult zebrafish (Bise et al. 2020). Interestingly, CM proliferation is activated after each injury (although this becomes less efficient over time), but collagen deposition is exacerbated by each injury and scar removal gradually fails, further suggesting that regenerative capacity has limits (Bise et al. 2020).

Zebrafish injury models have also been instrumental in clarifying the source of new heart muscle cells in a regenerative context. Several studies have exploited lineage-tracing methods to show that existing mature CMs are the source of new cardiac muscle and stem/progenitor cells have no significant involvement in this process (Jopling et al. 2010 and reviewed by Kikuchi 2015). Morphologically, the dividing zebrafish CMs change their contractile state in a manner similar to structural alterations observed to facilitate proliferation in murine cells, such as disassembly of sarcomeres, which may indicate conservation of the underlying processes required for CM proliferation (Ahuja et al. 2004; Jopling et al. 2010; and reviewed by Kikuchi 2015).

There is some CM proliferation in adult mammals and this likely arises from existing CMs (as in zebrafish); however, this occurs at a much lower frequency and the signature of the CMs retaining this proliferative ability has not yet been fully determined (Bergmann et al. 2009; Jopling et al. 2010; Senyo et al. 2013; Kikuchi 2015). Evidence has emerged in recent years to suggest that ploidy is an important factor. The majority of zebrafish CMs are mononucleated and diploid; however, these properties are lost in mammalian cells which become binucleated or polyploid soon after birth (Soonpaa et al. 1996; Kikuchi et al. 2010; Mollova et al. 2013; Ye et al. 2016). This hypothetical relationship has been further substantiated as the prevalence of mononuclear diploid CMs has been shown to correlate with functional recovery and CM proliferation after coronary artery ligation in mice and CM ploidy can generally predict regenerative potential across vertebrate species (Patterson et al. 2017; Hirose et al. 2019). Gonzalez-Rosa et al. have recently shown direct evidence of the importance of ploidy, as experimental polyploidisation of zebrafish CMs is sufficient to inhibit proliferative potential in this highly regenerative model, and a substantial proportion of diploid CMs (approximately 75%) is required to support regeneration, leading the authors to propose stimulation of the rare diploid cells in the human heart as a method to boost heart regeneration (González-Rosa et al. 2018).

The importance of CM replacement for complete regeneration to occur is clear, but the value of the interplay between these cells and their microenvironment is just beginning to be elucidated. The study of many different in vivo models of cardiovascular disease and injury is helping researchers look beyond CMs to the myriad of cells and processes that co-activate, co-ordinate and co-regulate regeneration of the heart. These processes include signalling mechanisms for cell recruitment (reviewed by Sanz-Morejón and Mercader 2020), phagocytic immune cell removal of debris (de Preux Charles et al. 2016; Lai et al. 2017; Bevan et al. 2020), fibroblasts laying down ECM (Simões et al. 2020) and MMP breakdown of ECM to permit the infiltration of new vasculature (Bellayr et al. 2009; Marín-Juez et al. 2016; Xu et al. 2019), which ultimately provide a supportive architecture for the invasion of new CMs. Further study of this complex interplay will be key to our understanding of the regenerative process as a whole.

## Zebrafish as a model for repair & regeneration

Numerous advantages of the zebrafish, such as their high fecundity, external fertilisation and the transparency of developing larvae, have been fundamental in establishing them as a valuable model system for vertebrate developmental biology (reviewed by Dooley 2000). Further to this, their genetic tractability has proven an adaptable asset during an extraordinary transitional period in genome editing, from early labour-intensive reverse genetics approaches like ENU-screening through to the now ubiquitous CRISPR-cas9 system (Koster and Sassen 2015; Sertori et al. 2016). As a non-mammalian model, there are genetic discrepancies, with the poor conservation of some alleles and duplication of approximately 20% of genes (Postlethwait 2000) sometimes making direct comparisons difficult. However, 70% of protein-coding human genes are related to genes found in zebrafish, and 84% of disease-related genes have a zebrafish equivalent (Howe et al. 2013) meaning they have become an important model of human disease (reviewed by Lieschke and Currie 2007). Antibody availability has historically been poor (though in recent years the prevalence of custom synthesis services and the popularity of the zebrafish as a model system has led to some improvements on this score), yet amenability to genome-editing has also facilitated the generation of numerous transgenic lines which are both a powerful research tool in their own right and partially compensate for the lack of commercially available antibodies.

In addition to these well-known characteristics, the key attribute for zebrafish in this research context is their near-unique ability to completely regenerate the adult heart post-injury, thus providing a cellular and molecular map for the processes from repair to regeneration (Poss 2002; Chablais et al. 2011; Schnabel et al. 2011; Gonzalez-Rosa et al. 2011; Bevan et al. 2020). As described above, three main models of cardiac injury have been described in adult zebrafish, (1) cardiac resection (Poss 2002), (2) cryoinjury (Chablais et al. 2011; González-Rosa et al. 2011; Schnabel et al. 2011) and (3) genetic ablation of CMs (Wang et al. 2011). The resection model, which involves the surgical removal of the ventricular apex, was used in the first landmark study describing the regenerative ability of the adult zebrafish heart (Poss 2002). First published in 2011, the cryoinjury model arguably provides the most representative model for the ischaemia-induced cell death associated with infarction, resulting in extensive scar formation (Chablais et al. 2011; González-Rosa et al. 2011; Schnabel et al. 2011; Bevan et al. 2020). Although these initial cryoinjury studies report some discrepancies in the “completeness” of the regeneration process, this is likely due to differences in the extent of the injury inflicted (20% ventricular area vs 25%, Chablais et al. 2011; González-Rosa et al. 2011, respectively), and overall, these studies largely correlate on the major events post-injury. Around the same time as the description of the cryoinjury model, it was shown that adult zebrafish could also recover from the loss of up to 60% of their CMs, ablated using a genetic Cre/LoxP-driven cytotoxic diptheria toxin A expression system (Wang et al. 2011). Although this system further highlights the remarkable regenerative capacity of the zebrafish and allows a less invasive method to study CM replacement, it is arguably the least representative of human cardiac damage as it is not localised, resulting in the random loss of CMs throughout the ventricle and, therefore, does not elicit the same targeted fibrotic, scarring and angiogenic responses, which are all important aspects of the repair to regeneration transition.

**Fig. 1:**
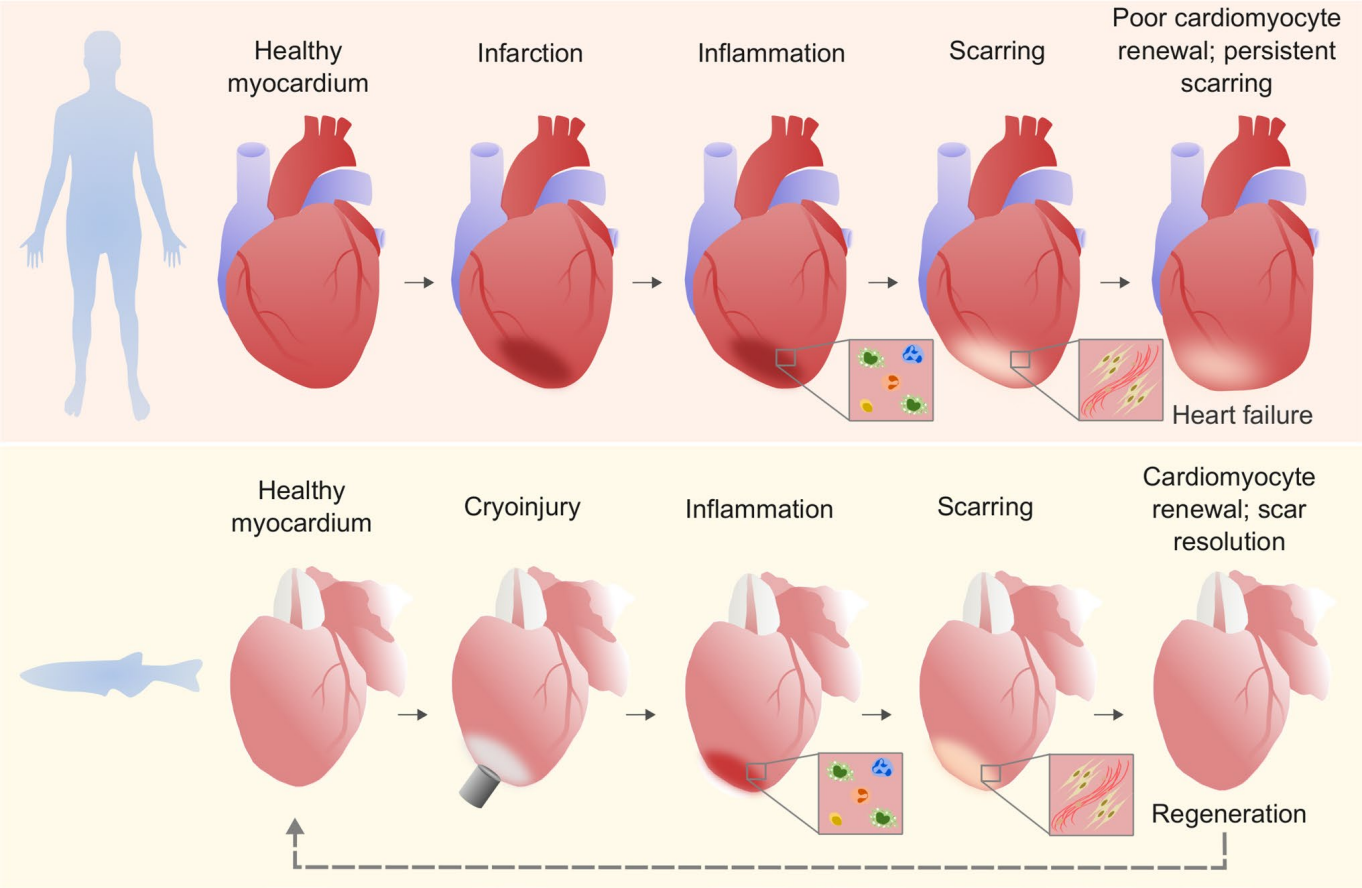
Human and zebrafish heart repair. Phases of repair/regeneration in human and zebrafish hearts post-injury, showing that initial inflammatory and scarring responses are similar, but the final stages diverge, with humans exhibiting persistent scar tissue and poor renewal of CMs. Zebrafish models of MI (e.g. cryoinjury shown here) exhibit a regenerative phase of scar resolution and CM proliferation, terminating in a return to healthy myocardium

It is important to note that following cardiac damage, such as MI (humans) or cryoinjury (zebrafish), the initial phases from injury to repair and scarring are conserved, further strengthening the value of the zebrafish as a powerful tool to understand the limiting factors preventing mammalian regeneration (Fig. 1 and reviewed by Giardoglou and Beis 2019). Both human and zebrafish repair involves an initial inflammatory phase (defined by the recruitment of immune cells and the clearance of cellular debris by phagocytosis) followed by a reparative phase characterised by deposition of collagen and other extracellular matrix (ECM) components and scar formation (Dobaczewski et al. 2011; Chablais and Jazwinska 2012). In humans, this collagenous matrix develops to form a mature scar that is never resolved; however, in zebrafish the deposed collagen is rapidly remodelled and replaced with new myocardium (Chablais et al. 2011; González-Rosa et al. 2011; Schnabel et al. 2011; Hortells et al. 2019).

Cryoinjury results in cell death within the ventricle wall (with apoptotic cells also detectable in the lumen of coronary vessels) which peaks at approximately 4 dpi and decreases progressively to below 0.5% at 60 dpi (Chablais et al. 2011; González-Rosa et al. 2011; Schnabel et al. 2011). This apoptotic peak is concomitant with the initial inflammatory response and the commencement of neovascularisation with existing coronary vessels sprouting into the injury area (González-Rosa et al. 2011; Marín-Juez et al. 2019). Extensive fibrin accumulation in the injury areas is also seen at 4 dpi (Chablais et al. 2011; Schnabel et al. 2011) but is mostly eliminated by 14–21 dpi (Chablais et al. 2011; González-Rosa et al. 2011; Schnabel et al. 2011). Extensive CM (and other cell) proliferation is observed during these initial phases of the injury response, peaking within the first week (Chablais et al. 2011; González-Rosa et al. 2011; Schnabel et al. 2011). By 21 dpi, vessel coverage of the injured area is complete, and this re-vascularisation of the injured area is so rapid that a mere 40 days after injury the vessels are indistinguishable between controls and injured hearts (González-Rosa et al. 2011; Marín-Juez et al. 2019).

The deposition, remodelling and maturation of the collagen-rich scar are the final phase in the mammalian repair process; however, the resolution of this tissue occurs rapidly in the zebrafish, with scar clearance and regeneration of the cardiac tissue being completed within 60–130 dpi depending on the size of the initial injury (Bevan et al. 2020; Schnabel et al. 2011; Chablais et al. 2011; González-Rosa et al. 2011; and reviewed by Dittrich and Lauridsen 2019). Interestingly, comparative analyses between cryoinjury and resection injury responses indicate differences in the degree of apoptosis, inflammation and scarring, further highlighting variation between injuries (Chablais et al. 2011; Simões et al. 2020). This regenerative capacity in an adult in vivo system provides an unparalleled opportunity to study the molecular and cellular processes of regeneration in an intact environment.

## Emerging roles for inflammatory populations

**Fig. 2:**
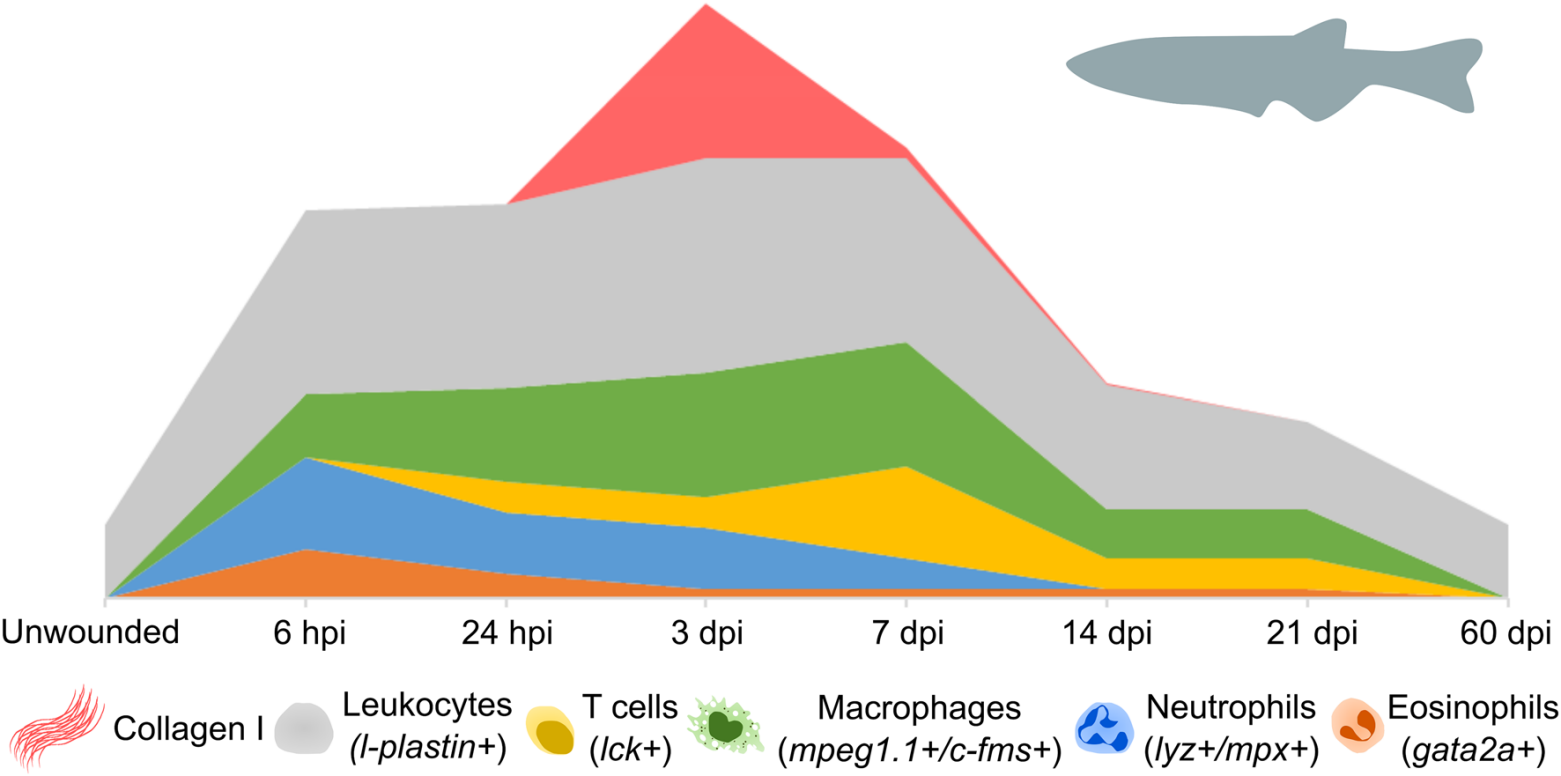
Immune cell population and ECM dynamics during zebrafish cardiac repair/regeneration. Diagram adapted from data published by Bevan et al. (2020), showing relative waves of immune cell populations and collagen (specifically Collagen I) deposition as detected over a 60-day time period following cardiac cryoinjury. A colour-coded legend, including markers used to define the relative populations, is shown below the graph

There has been a recent shift to focus on inflammation as a crucial aspect of the regeneration process. Zebrafish have many innate and adaptive immune cell populations which are analogous to mammals and can be found in similar ratios within the healthy and injured zebrafish heart (Herbomel et al. 1999; Wittamer et al. 2011; Dee et al. 2016; Bevan et al. 2020). Our group and others have described the systematic recruitment and expansion of innate and adaptive immune cell populations and have begun to uncover their roles in the adult zebrafish heart during the characteristic phases of the repair and regeneration timeline (de Preux Charles et al. 2016; Lai et al. 2017; Hui et al. 2017; Sanz-Morejón et al. 2019; Bevan et al. 2020; Simões et al. 2020) (Fig. 2). This has shown the direct parallels that exist between zebrafish and mammalian cardiac repair, with timely induction and resolution of the inflammatory response being essential to achieve normal regeneration in the zebrafish heart and to regulate many other aspects of repair, including revascularisation, CM proliferation and scar deposition and resolution (Bevan et al. 2020; Simões et al. 2020). Recent observations have also illustrated that without tight regulation of the inflammatory response, excessive immune cell accumulation is inhibitory to regeneration, regardless of the presence of proliferating cells and the ECM (Xu et al. 2019). This demonstrates that inflammatory cells have critical roles in coupling CM proliferation and scar resolution, both of which are central to an efficient regenerative outcome. A thorough characterisation of immune cell types during all the repair and regeneration stages and a full understanding of the differences between zebrafish and mammals will be essential to understanding their diverse roles after injury. Here we introduce the main immune cell types that have been studied in the context of cardiac regeneration.

### Granulocytes

Granulocytes are key components of the innate immune system and include neutrophils and eosinophils (reviewed by Lin and Loré 2017) whose roles and dynamics following cardiac injury in the adult zebrafish are beginning to be defined (Lai et al. 2017; Xu et al. 2018; Bevan et al. 2020).

Neutrophils are amongst the “first responders” to tissue damage and are rapidly mobilised and recruited to the cryoinjured heart from 6 hours post injury (hpi) (Bevan et al. 2020). Neutrophil numbers peak during the first 24 hpi (Fig. 2), during which time they are major contributors to the pro-inflammatory phase of repair (Lai et al. 2017; Xu et al. 2018; Bevan et al. 2020). In addition to the phagocytosis of necrotic tissue, recruited neutrophils secrete pro-inflammatory mediators, reactive oxygen species (ROS) and cytokines which recruit additional immune cells and promote myofibroblast differentiation and angiogenesis, which are essential for later stages of regeneration (Lai et al. 2017; Xu et al. 2018; Bevan et al. 2020). However, the release of ROS by neutrophils, as well as infiltrating monocytes, can cause further tissue necrosis and scarring (Bonaventura et al. 2019). Neutrophil retention has been shown to prolong the inflammatory period leading to delayed scar regression and reduced CM proliferation in heart injury models (Robertson et al. 2014; Lai et al. 2017; Xu et al. 2019), therefore, timely cessation of the neutrophil response is essential for the regenerative outcome observed in zebrafish.

Eosinophils are also rapidly recruited to the injured zebrafish heart and remain elevated from 7–21 dpi (Bevan et al. 2020). Despite this, functional studies have yet to be performed for this cell type in zebrafish so further investigation will be required to elucidate their role in coordinating repair and regeneration.

### Macrophages

The importance of diverse macrophage functions during wound healing has been well-described in both reparative and regenerative organisms (Nahrendorf et al. 2007; Mirza et al. 2009; Lavine et al. 2014; Petrie et al. 2015; Nguyen-Chi et al. 2017; Morales and Allende 2019). This includes phagocytic and pro-inflammatory activity in the initial stages of repair as well as the reversal of the inflammatory environment to homeostatic conditions (Nguyen-Chi et al. 2017; Bevan et al. 2020). The functional repertoire of macrophages is also ever-expanding, with recent publications identifying novel roles for macrophages in wound angiogenesis (Gurevich et al. 2018), electrical conductance during cardiac homeostasis (Hulsmans et al. 2017) and collagen deposition after heart injury in both murine and zebrafish models (Simões et al. 2020). Macrophages have historically been broadly grouped by function and activation state as either “M1” or “M2”, with M1 macrophages thought of as pro-inflammatory and anti-microbial, and M2 macrophages considered to be pro-reparative, with roles in tissue re-modelling, immune regulation, matrix deposition and phagocytosis (reviewed by Lee 2019). However, it is increasingly evident that the diverse, and sometimes surprising, roles being ascribed to cardiac macrophages are coordinated by subsets with highly heterogenous phenotypes, transcriptional profiles and activation states, which are much more transient than the traditional M1 or M2 classification. This M1/M2 nomenclature is now largely seen as an oversimplification, with macrophages more likely to have a spectrum of activation states that inform their function. This plasticity of macrophage phenotype and function is largely controlled by the tissue microenvironment, but is also determined by cellular origin, with tissue resident and monocyte-derived populations shown to be distinct populations (He et al. 2018; Ferrero et al. 2018), which, in turn, is likely to affect their contribution to regeneration and scarring (Lavine et al. 2014).

The genetic tools and imaging capabilities presented by the zebrafish are unveiling important insights into the complexities of these populations during adult heart regeneration. Tracking of global macrophage populations during zebrafish heart regeneration indicates that there is an accumulation of macrophages within the ventricle at 3–7 dpi, which is largely resolved by 14 dpi (Fig. 2) (Lai et al. 2017; Bevan et al. 2020; Simões et al. 2020). To date, studies of macrophages in zebrafish have been assisted by the use of select transgenic reporters (namely driven from *mpeg1.1* (Ellett et al. 2011) and *csf1ra* (Gray et al. 2011) promoters). These can be combined with transgenics that indicate the inflammatory activation of these cells (currently using *tnfa* (Nguyen-Chi et al. 2017) or *il1b* (Ogryzko et al. 2019) promoters) to further categorise these cells by their inflammatory phenotype. To this end, we have recently shown that distinct waves of macrophage populations (pro-inflammatory and pro-resolutionary) respond to cardiac injury, and interference with these populations leads to prolonged scar retention (Bevan et al. 2020). Similarly, others have shown that delayed monocyte recruitment leads to increased scar area, further demonstrating their requirement for correct injury resolution (Xu et al. 2018). Manipulation of macrophage dynamics also prolongs neutrophil activity and impairs normal neovascularisation, CM replacement and scar removal (De Preux Charles et al. 2016; Lai et al. 2017; Bevan et al. 2020). This suggests that macrophages are important for controlling the inflammatory environment in wounded tissue, partly by neutrophil clearance, and fostering an environment for efficient angiogenesis and CM proliferation.

However, future work is necessary to further delineate the macrophage subsets that are responsible for these processes in zebrafish. Moreover, the recent finding that *mpeg1.1*, which has been ubiquitously used to label macrophages in zebrafish, is also expressed in a sub-population of B-lymphocytes and natural killer-like cells in adult zebrafish (Moyse and Richardson 2020; Ferrero et al. 2020) demonstrates the need to expand and diversify macrophage markers. Macrophage phenotype and activation states are emerging as key regulators of these opposing processes (Bevan et al. 2020), yet as the importance of the immune response in repair and regeneration becomes clear, the need to fully understand the roles of immune cells in these processes is ever greater. Stratification of these cells will therefore be essential to fully appreciate their contribution to regeneration.

### Lymphocytes

The relationship between the adaptive immune system and regeneration has been a source of debate, as organisms with more advanced immune systems generally have reduced regenerative capacity (Hui et al. 2017; Sattler et al. 2017). As a result, the roles of neutrophils and macrophages in wound healing and regeneration have been extensively studied, yet the contribution of adaptive immune cells is less well understood, particularly with respect to cardiac regeneration. However, it is becoming increasingly apparent that many regenerative organisms, including zebrafish, possess sophisticated adaptive immune systems, justifying the need to study these cells during regeneration.

Lymphocytes of the adaptive immune system, such as T cells, coordinate antigen-specific immunity. There are many subsets of T cells with varying functions, such as tumour and viral immunity, cytokine secretion and the establishment of humoral responses. A further subset, CD4+ FOXP3+ T regulatory cells (Tregs), are important for suppressing the inflammatory response by secretion of anti-inflammatory cytokines such as IL-10 (Kasheta et al. 2017). These T cell populations have been shown to have diverse and opposing roles in mouse models of MI, with known functions in wound healing, inflammation and regulation of CM numbers (reviewed by Epelman et al. 2015; Yang et al. 2006; Varda-Bloom et al. 2000); therefore, further investigation is warranted to define T cell dynamics in response to cardiac injury.

Adult zebrafish possess analogous T cell populations to the major subsets identified in mammals, namely CD4+ T helper cells (Langenau and Zon 2005; Dee et al. 2016) which include Tregs (Kasheta et al. 2017) and also γδ T cells (Wan et al. 2017). They are therefore a fantastic model system to study the role of T cells in cardiac repair and regeneration. Recently, Hui et al. (2017) described the importance of Tregs in the promotion of CM proliferation and in the control of macrophage activation state post-injury (Hui et al. 2017). Indeed, a comparative study of zebrafish and a closely related but non-regenerative teleost fish, medaka, has highlighted T cell-specific pathways in effective cardiac regeneration in response to cryoinjury. Zebrafish were shown to have strong upregulation of genes involved in T cell proliferation and B cell receptor signalling unlike medaka (Lai et al. 2017). This suggests that lymphocytes have significant involvement in cardiac repair and regeneration that is yet to be fully elucidated.

Adult zebrafish also possess populations of B cells (Page et al. 2013) and natural killer-like cells (Carmona et al. 2017), and recent studies demonstrate that a proportion of these populations are labelled by *mpeg1.1* transgenics (Moyse and Richardson 2020; Ferrero et al. 2020). Indeed, we have shown that these *mpeg1.1* expressing lymphocytes respond to cardiac cryoinjury in a different temporal manner to *mpeg1.1*+ macrophages suggesting that they could play a role in repair or regeneration responses (Moyse and Richardson 2020). Future studies will hopefully reveal more about the role these adaptive immune cell populations are playing within a beneficial, regenerative cardiac microenvironment.

## Extracellular matrix composition and function

The formation of a mature scar which is not resolved majorly inhibits cardiac function post-MI in mammals (reviewed by Hortells et al. 2019) and so the composition of the ECM and the cells which regulate it should also be considered important players in the regenerative process.

The myocardial ECM is an intricate protein network, composed primarily of collagen, and with a tripartite organisation that surrounds CMs (the endomysium), defines major cardiac tissue bundles (the perimysium) and encapsulates the whole cardiac muscle (the epimysium) (reviewed by Frangogiannis 2017). It surrounds the heart, providing a scaffold and maintains the architecture of the tissue. In both mammals and zebrafish, cardiac fibroblasts are the major contributory cell type in ECM production. Normally quiescent, interstitial fibroblasts respond rapidly to stimuli such as injury or hypoxia, re-entering the cell cycle, synthesising ECM proteins, moderating cell–cell communication and producing activated fibroblasts and myofibroblasts (Moore-Morris et al. 2014; Kanisicak et al. 2016; Ivey et al. 2018).

In mammals, the cardiac ECM is thought to be composed mainly of collagen I (> 85%), with other collagens, fibronectin, glycosaminoglycans (GAGs) and proteoglycans comprising the rest of the matrix (reviewed by Frangogiannis 2017). The exact composition of the zebrafish ECM is unknown; however, comparison with murine samples has revealed that zebrafish ECM contains significantly less collagen and more elastins and GAGs (Chen et al. 2016). Additional information on the ECM composition has been gleaned from immunodetection assays, which have shown the presence of several matrix proteins during zebrafish cardiac regeneration, including structural proteins such as non-fibrillar Collagen XII and fibrillar Collagen I, the adhesive protein Fibronectin and the de-adhesive protein Tenascin C (Chablais and Jazwinska 2012; Wang et al. 2013; Marro et al. 2016). Decellularised cardiac ECM from zebrafish has been shown to have a significant proliferative effect on human cardiac precursor cells and enables endogenous regeneration of mouse heart tissue post-MI (Chen et al. 2016). These clear differences in zebrafish vs mammalian ECM highlight its importance in mediating regeneration and demonstrate the merit in continued research into the exact manner in which ECM components are generated and resolved.

In adult mammals, cardiac fibroblasts are required for scar formation, and their contribution to the ECM profile post-injury can influence CM proliferation and hypertrophy, but ultimately there is low CM proliferation, limited scar resolution and thus fibroblasts transition to matrifibrocytes and a mature scar is formed that reduces cardiac function (reviewed by Hortells et al. 2019). However, in zebrafish, endocardial fibroblasts synthesise ECM collagen post-injury and are inactivated during scar resolution. Though limiting the fibrotic response via ablation of *col1a2-*expressing cells does not affect regeneration, it does reduce the number of proliferating CMs, hence both fibroblasts and the ECM are necessary to stimulate CM proliferation and regeneration (Sánchez-Iranzo et al. 2018). Recent work has also revealed that it is not only myofibroblasts that contribute to fibrosis during heart repair, but macrophages also deposit collagen post-injury in both zebrafish and mouse models (Simões et al. 2020). This newly identified function for macrophages further highlights the fascinating potential of immune cells, and also the extent to which their importance is still incompletely understood.

## Extracellular vesicles, cilia and signalling networks

While looking outside of the traditional focus on CM proliferation, it is important to consider not just which cell types are implicated, but how they are recruited and activated. One under-explored aspect of cell–cell communication is the topical field of extracellular vesicle (EV) research. These particles, bound by a lipid bi-layer, are produced and released by most cell types and can be broadly divided into three classes depending on their biogenesis: exosomes, microvesicles and apoptotic bodies (Van Niel et al. 2018; Caruso and Poon 2018). EVs have been isolated from pericardial fluid from patients undergoing cardiac surgery (Kuosmanen et al. 2015; Beltrami et al. 2017) and have been implicated in the progression of cardiovascular disease (Wang et al. 2014; Emanueli et al. 2015), but are also thought to have roles in mediating cell–cell communication between different cell types within the heart, including CMs, fibroblasts and immune cells (Bang et al. 2014, 2015; Todorova et al. 2017; Zhou et al. 2019). Further to this, there is emerging data that suggest there may be EV populations that are cardioprotective and promote angiogenesis (Emanueli et al. 2015; Todorova et al. 2017; Zhou et al. 2019).

Despite this mounting evidence showing the importance of EVs, what we know thus far has been largely derived from in vitro studies (Van Niel et al. 2018); however, novel methods are being developed to address the complexity and heterogeneity of endogenous EVs. Our laboratory has recently developed one such tool, combining the in vivo nature of the zebrafish model with a cell–membrane tagged fluorophore approach to facilitate future investigations of endogenous cell-type-specific EV populations (Scott et al. 2019). This approach coupled with the cardiac cryoinjury model demonstrates dynamic changes in EV production post-injury and has potential to unlock the role of EVs in cell communication during repair and regeneration.

Another sub-cellular component with potential implications in this area of cell–cell communication is the cilium. Research into the primary cilium and its role in different disease states has also expanded exponentially in recent years. Initially thought to be no more than an evolutionary vestige, primary cilia are now known to be present on the majority of cells and have been revealed to be crucial signalling antennae, sending and receiving information across cell and tissue types. Primary cilia have roles in fundamental cell processes like proliferation, differentiation and regulation of the cell cycle (Delling et al. 2013; Yuan et al. 2016) and respond to a wide range of stimuli including growth factors (Schneider et al. 2005) and glucocorticoids (Wang et al. 2012) as well as mechanical stress and flow (Hierck et al. 2008; Nauli et al. 2013). It is thus unsurprising that cilia are becoming a subject of interest in the field of cardiovascular research, with a recent publication detailing their previously unknown requirement for cardiac fibrosis following MI in both zebrafish and human tissue (Villalobos et al. 2019).

Sub-cellular components such as cilia and EVs have historically been challenging to study due to limitations in imaging and analysis; however, novel methods and improved technology are revealing that these miniscule actors play crucial roles in regulating complex and dynamic microenvironments in vivo and new studies are highlighting the zebrafish as an ideal model system in which to study them.

During regeneration, CM proliferation must proceed in concert with the removal of the deposited scar tissue. The damaged tissue is subject to remodelling and re-organisation as it grows, and these processes are orchestrated via a combination of adhesion, migration and signalling events (reviewed by Sanz-Morejón and Mercader 2020). The roles and consequences of signalling pathways in these processes are complex and manifold, with CMs at the injury site exposed to signals including Pdgf, RA, Igf, Shh, Tgfβ ligands, BMP and Nrg1, which are secreted from the epicardium, epicardium-derived cells, endocardial cells and circulating cells (reviewed by González-Rosa et al. 2017). NF-κB signalling is also required for CM dedifferentiation and proliferation, as well as epicardial regeneration (Karra et al. 2015), and Notch signalling (via* serpine1*) is required for endocardial and myocardial proliferation (Münch et al. 2017). Hedgehog signalling ligands have been determined to be essential for controlling epicardial migration and are produced by smooth muscle cells from the bulbus arteriosus (Wang et al. 2015). The important publications in this area are so numerous that a comprehensive discussion of these processes goes beyond the focus of this publication and have been discussed recently elsewhere (González-Rosa et al. 2017). However, the versatility of the zebrafish model has led to the identification of important loci in the control of scar deposition/resolution and other regenerative processes, and so some specific examples of newly-identified genetic players and their contributions to the understanding of the complex signalling cascades required for regeneration are featured here.

It has recently been shown that the transcription factor Runx1 is a key regulator of both scar deposition and degradation, as well as proliferation of the myocardium (Koth et al. 2020). Not only was Runx1 seen to be specifically upregulated in endocardial cells and thrombocytes in the injury region, which in turn induced expression of smooth muscle and collagen genes during zebrafish heart regeneration, but targeted mutation and the subsequent absence of *runx1* resulted in an increase in survival and proliferation of the myocardium and overall heart regeneration and decreased fibrosis resulting from a reduction in myofibroblast formation and upregulation of the fibrin degeneration pathway (Koth et al. 2020). This discovery is particularly interesting given that Runx1 is upregulated in CMs post-injury in a number of species, both regenerative and not, and presents a potential therapeutic target that could be manipulated to induce endogenous human heart regeneration (Gattenlöhner et al. 2003; Kubin et al. 2011; Eulalio et al. 2012; Górnikiewicz et al. 2016; Goldman et al. 2017; Koth et al. 2020).

In terms of CM proliferation, there have also been some interesting observations that help set the process apart in zebrafish, identifying differences in the effects of signalling mechanisms. Increased Yap/Taz in murine CMs has been shown to augment the renewal of cardiac tissue following ischaemia, yet CM proliferation in zebrafish *yap* mutants is not altered following cryoinjury, contrary to reports from the murine study; however, the collagen composition of scars is decreased and the injury size observed is larger, indicating that Yap is required for scar formation during zebrafish heart repair, indirectly mediating ECM deposition (Leach et al. 2017). The role of nerve cells in CM proliferation is both conserved and disparate between the regenerative models of zebrafish and neonatal mice. Cardiac re-innervation following injury is necessary in both neonatal mice and zebrafish, with Nrg1 capable of stimulating CM proliferation after injury (Mahmoud et al. 2015). Interestingly, however, the source of Nrg1 is not conserved, as murine production of Nrg1 occurs in nerve cells but it derives from perivascular cells in zebrafish, despite the conserved requirement for nerve cells in both systems (Gemberling et al. 2015; Mahmoud et al. 2015). Exploration of these differences between regenerative and non-regenerative, in addition to vertebrate and invertebrate species may be key to finding targets for future genetic or pharmaceutical therapies. A study of the revascularisation that occurs post-injury has also revealed that there are two kinds of coronary sprouting that have separate mechanistic controls, with superficial sprouting (within the regenerating epicardium) regulated by chemokines cxcl12 and cxcr4, whereas intraventricular sprouting (towards the activated endocardium) is controlled by vegfa. These then combine to provide a scaffold for CM repopulation of the injured heart; however, inhibition of early revascularisation via a dominant negative form of *vegfaa* reduces CM proliferation and inhibits regeneration (Marín-Juez et al. 2019). Consistently, knockout of *cxcr4a* also inhibits regeneration (Harrison et al. 2015). This type of data can help compare and highlight important differences in the regulatory pathways that are controlling the regenerative response.

This cache of valuable genetic data is not limited to zebrafish however, as recent studies in another teleost fish have shown. Comparative genetics in a study of Mexican cave fish *Astyanax mexicanus* has also yielded a wealth of data, following the striking observation that a surface-dwelling population of these fish are capable of complete cardiac regeneration after injury yet their cave-dwelling counterparts are not, despite being of the same species. Independent evolution of the populations has provided an unparalleled system for direct comparison of their respective scarring and regenerative responses, and the ability to identify key genetic players in the capacity for heart regeneration without the complications of presented by interspecies comparisons. The authors were able to identify *lrrc10* as a gene specifically upregulated in surface fish (regenerative) compared to cavefish (non-regenerative), and a complementary zebrafish knockout model showed impaired heart regeneration despite unaffected proliferation of the CMs (Stockdale et al. 2018). This further supports the importance of looking beyond CM proliferation to consider all the components of the complex microenvironment. Further to this, quantitative trait analysis allowed the authors to identify three genomic loci that seem to be linked to the magnitude of heart regeneration (Stockdale et al. 2018).

Though regeneration mechanisms are generally believed to be tissue-specific (reviewed by Beffagna 2019), there is also evidence from zebrafish to suggest that there may in fact be some common regulatory elements, with the discovery by Pfefferli and Jaźwińska of *careg,* a regulatory element that contains a ctgfa upstream sequence that is transiently activated but common to both heart and fin regeneration, and induced by TGF-β/Activin-β signalling (Pfefferli and Jaźwińska 2017).

Though in no way comprehensive, the above-mentioned studies show how the zebrafish can provide valuable insights into the signalling pathways and communication networks involved in a regenerative vertebrate system. By comparing these to non-regenerative systems, this will help to elucidate conserved regulatory elements but also identify key differences that may be critical to achieving a regenerative phenotype.

## Looking beyond cardiomyocytes

**Fig. 3:**
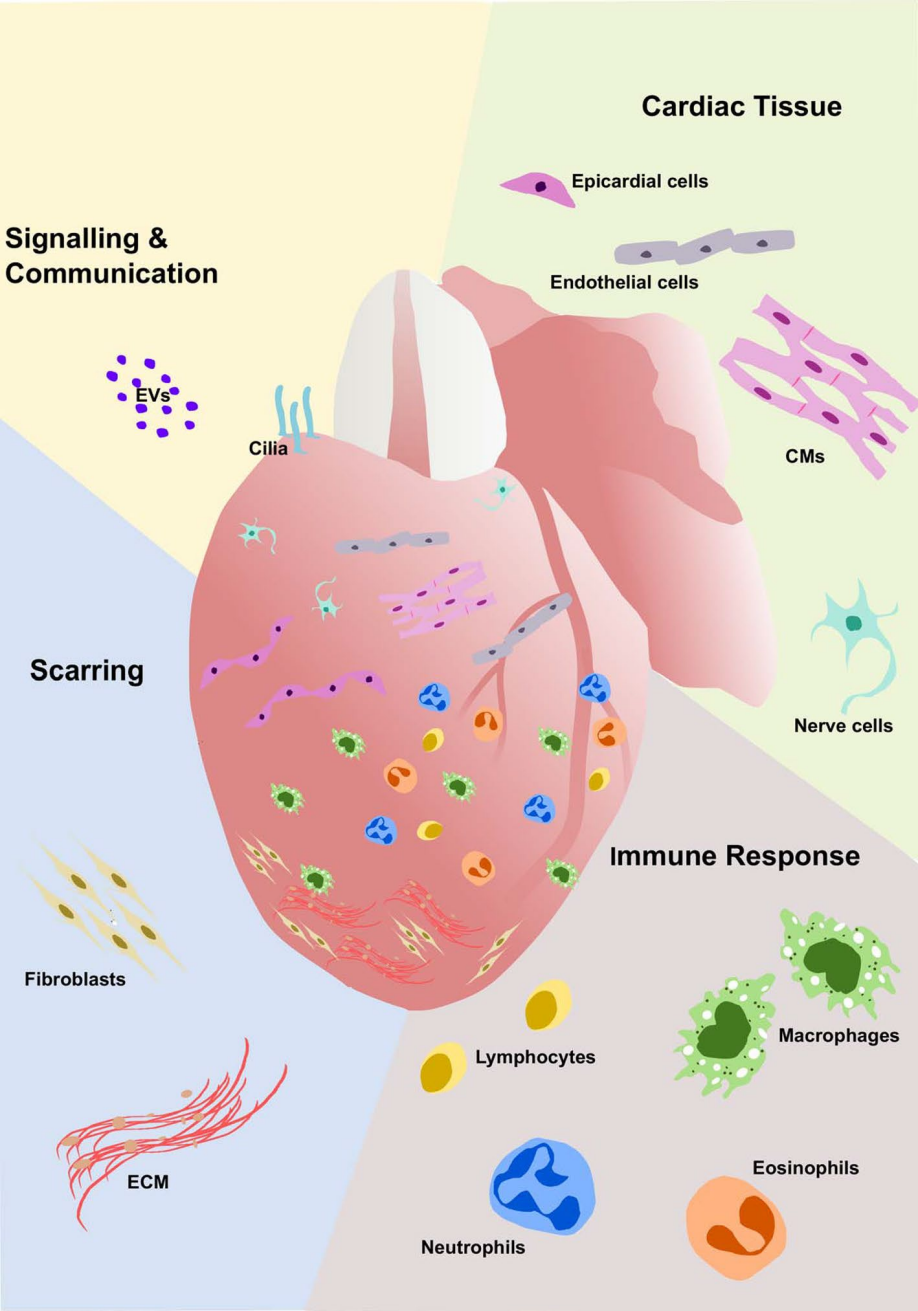
Heart repair and regeneration requires a concerted effort by many different cell types. Representation of the numerous cell populations required throughout heart repair and regeneration in the adult zebrafish. Sample publications detailing the roles of these different cell types are listed in Table 1

As we look beyond CMs, the adverse microenvironment is full of complex cues that are not yet fully understood. The cells of the immune response alone have an ever-increasing functional roster in both repair and regeneration, including emerging roles for macrophages in wound angiogenesis (Gurevich et al. 2018) and collagen deposition (Simões et al. 2020). Additionally, studies are describing multiple crucial roles for other cell types within the heart including epicardial cells (reviewed by Masters and Riley 2014; Cao and Poss 2018), endothelial cells (Marín-Juez et al. 2016), nerves (Mahmoud et al. 2015), fibroblasts (Sánchez-Iranzo et al. 2018) and lymphatic cells (Gancz et al. 2019; Harrison et al. 2019; Vivien et al. 2019) (summarised in Fig. 3 and Table 1). The importance of all the steps in the cascade should not be underestimated, as each additional piece of the puzzle contributes to rebuilding the heart. The interplay between the physical and molecular processes that orchestrate to regenerate the heart is still incompletely understood; however, the zebrafish presents an invaluable system to study the interplay of these different cells and processes. This regenerative adult in vivo model is amenable to high-throughput genetic editing approaches and live-imaging is unparalleled in its potential to unravel the complexities of the therapeutic holy grail that is human heart regeneration.

**Table 1:** Components of the cardiac microenvironment important for regeneration

**Immune cells**	
Macrophages	Bevan et al. (2020); Ellett et al. (2011); Ferrero et al. (2018); Gray et al. (2011); He et al. (2018); Lai et al. (2017); Lavine et al. (2014); Nguyen-Chi et al. (2017); Ogryzko et al. (2019); de Preux Charles et al. (2016); Simões et al. (2020); Xu et al. (2018)
Neutrophils	Bevan et al. (2020); Lai et al. (2017); Robertson et al. (2014); Xu et al. (2018)
Eosinophils	Bevan et al. (2020)
Lymphocytes	Bevan et al. (2020); Carmona et al. (2017); Dee et al. (2016); Hui et al. (2017); Kasheta et al. (2017); Lai et al. (2017); Langenau and Zon, (2005); Moyse and Richardson (2020); Page et al. 2013; Wan et al. (2017)
**Cardiac tissue**	
Cardiomyocytes	Bise et al. (2020); Chablais et al. (2011); González-Rosa et al. (2011); González-Rosa et al. (2018); Jopling et al. (2010); Kikuchi et al. (2010); *Kikuchi, (2015); Schnabel et al. (2011)
Fibroblasts	Sánchez-Iranzo et al. (2018)
Endothelial/endocardial cells	Münch et al. (2017); Marín-Juez et al. (2019)
Epicardial cells	*Cao and Poss (2018); Marín-Juez et al. (2019); *Masters and Riley (2014); Schnabel et al. (2011); Wang et al. (2013); Wang et al. (2015)
Nerve cells	Mahmoud et al. (2015)
Lymphatic cells	Gancz et al. (2019); Harrison et al. (2019); Vivien et al. (2019)
**Scarring**	
ECM	Chablais and Jaźwińska (2012); Chen et al. (2016); Marro et al. (2016); Sánchez-Iranzo et al. (2018); Simões et al. (2020); Wang et al. (2013)
**Cilia and extracellular vesicles**	
EVs	Scott et al. (2019)
Cilia	Villalobos et al. (2019)

Sample publications detailing the role of various important components of the microenvironment required for zebrafish heart regeneration are listed here. This table is not exhaustive but includes some of the major publications discussed throughout this review

An asterisk (*) denotes review articles

## Summary

In this review, we have covered the advantages of the zebrafish model for cardiac regeneration research with a particular focus on the roles of different cell types within the cardiac microenvironment. Many of these roles are being uncovered by studying zebrafish and their remarkable natural regenerative ability. There is no doubt that the recovery of CM number is crucial to the regeneration of the heart and the return to full functional capacity, but studies in zebrafish and other regenerative models are revealing the plethora of signalling and other functions delivered by immune cells, epicardial cells, nerves, fibroblasts and endothelial cells that are crucial to supporting this regenerative outcome. In our minds, future therapeutic approaches will need to incorporate the protection or replacement of these other cell types, as well as CMs, to be effective. Groups studying the zebrafish are leading the way in this investigation into the complex cardiac microenvironment.
